# Orthologs of Human-Disease-Associated Genes in Plants Are Involved in Regulating Leaf Senescence

**DOI:** 10.3390/life13020559

**Published:** 2023-02-16

**Authors:** Hou-Ling Wang, Weilun Yin, Xinli Xia, Zhonghai Li

**Affiliations:** National Engineering Research Center for Tree Breeding and Ecological Restoration, College of Biological Sciences and Technology, Beijing Forestry University, Beijing 100083, China

**Keywords:** disease-related genes, leaf senescence, ortholog genes, protein–protein interactions, regulatory network

## Abstract

As eukaryotes, plants and animals have many commonalities on the genetic level, although they differ greatly in appearance and physiological habits. The primary goal of current plant research is to improve the crop yield and quality. However, plant research has a wider aim, exploiting the evolutionary conservatism similarities between plants and animals, and applying discoveries in the field of botany to promote zoological research that will ultimately serve human health, although very few studies have addressed this aspect. Here, we analyzed 35 human-disease-related gene orthologs in plants and characterized the genes in depth. Thirty-four homologous genes were found to be present in the herbaceous annual plant *Arabidopsis thaliana* and the woody perennial plant *Populus trichocarpa*, with most of the genes having more than two exons, including the ATM gene with 78 exons. More surprisingly, 27 (79.4%) of the 34 homologous genes in *Arabidopsis* were found to be senescence-associated genes (SAGs), further suggesting a close relationship between human diseases and cellular senescence. Protein–protein interaction network analysis revealed that the 34 genes formed two main subnetworks, and genes in the first subnetwork interacted with 15 SAGs. In conclusion, our results show that most of the 34 homologs of human-disease-associated genes in plants are involved in the leaf senescence process, suggesting that leaf senescence may offer a means to study the pathogenesis of human diseases and to screen drugs for the treat of diseases.

## 1. Introduction

The plant and animal kingdoms comprise highly contrasting life forms. The genome sequence of eukaryotes provides a powerful means and strategy that can be used to uncover the genetic basis of differences between organisms, and their detailed functional characterization can be further explored. Moreover, flowering plants have ancestral features that are conserved between plants and animals [1]. *Arabidopsis thaliana* is an ideal model for flowing plant genome and gene functional analysis because of its small nuclear genome as well as its small size, short generation time and large number of offspring. Multiple plant-specific biological processes have been uncovered including development [2,3], gene network regulation [4,5], metabolism [6,7], photomorphogenesis [8,9], immunity [10,11], DNA repair [12,13], environment responses, biotic and abiotic stress signaling [14,15,16,17,18]. The great number of advances made in *Arabidopsis* research not only support plant biology studies but also shed light on agricultural breeding, and directly contribute to comparative genomics, bioinformatics, molecular genetics, evolutionary biology, combinatorial chemistry and chemical genetics research. Most importantly, the progress of research in *Arabidopsis* supports the development and application of medicine.

By BLAST and comparing the list of human disease genes [19] with the *Arabidopsis* genome, we can observe that most of the functional and representative protein domains are conserved in similar proportions for both humans and *Arabidopsis* [1]. In fact, 48.1% (139/289) of human disease genes show hits for *Arabidopsis* with E < 10^−10^ using the BLASTP threshold method. In total, 36 (12.5%) had scores better than E < 10^−70^, including 17 that were highly conserved in *Arabidopsis* compared to yeast, *Drosophila* or *C. elegans* [1]. As multiple non-communicable human diseases and their severity are closely related to certain underlying risk factors, as well as ageing, cellular senescence is gaining increasing attention and is considered to be a potential target in treating human diseases [20]. Cellular senescence can be triggered by multiple factors, such as inflammation, oncogenes, reactive metabolites, mitogens, proteotoxic stress, DNA damage and damage-associated molecular patterns [21,22]. DNA damage is the key inducer of cellular senescence [23]. Dysfunction, mutations, radiation, and alkylating agents can cause DNA damage, and most DNA damage-related genes are senescence-associated genes, such as ataxia telangiectasia mutated (ATM) [24,25]. The p53 pathways is central for the inducers triggered cellular senescence phenotype, and p53 is a key mediator of adipogenesis and the glucose response gene [26], known as its cell cycle- and senescence-associated regulator property. Reducing the expression of p53 can delay cellular senescence but might also increase the risk of cancer [27]. The senescence-associated secretory phenotype (SASP) is one of the significant hallmarks. The ‘jumping genes’ (transposons) contribute to the SASP, as they are produced within senescent cells and re-inserted into the senescent cell DNA [28,29]. Senescence-targeting interventions represent promising strategies for clinical use across the lifespan [30], and ‘senolytic’ drugs, which can kill senescent cells or inhibit ASAP, can be was applied [27].

Plant senescence shares partial similarity with human senescence. Almost all plants undergo cell, tissue and organ senescence before they eventually die. Plant fitness is founded on timely senescence, which is considered as an evolutionarily acquired process [31]. During senescence, plants integrate information related to their developmental age with multiple internal and external signals via intricate regulatory pathways [32,33]. Leaf senescence, which has been widely studied, is the most representative and well-visualized type of senescence in plants. Currently, 31,214 genes and 1037 mutants of 86 species related to leaf senescence were summarized [34], and complex regulating signaling network analysis was conducted 30]. The circadian rhythm and aging clock, defined as endogenous time-keeping mechanisms which are closely related to human disease [35], are also linked to leaf senescence via cross-regulatory networks. Leaf age affects the circadian clock, and a shorter circadian period was observed in old leaves, as it is regulated by the circadian clock oscillator TIMING OF CAB EXPRESSION1 (TOC1) [36]. CIRCADIAN CLOCK–ASSOCIATED 1 (CCA1) is an evident regulator of leaf senescence, and the loss of function of CCA1 leads to early leaf senescence [37]. Specifically, CCA1 targets the leaf senescence master regulator ORE1 by repressing its expression and activates the expression of the chloroplast maintenance gene GLK2. ORE1 integrates the circadian clock with age-dependent senescence through PSEUDO-RESPONSE REGULATOR9 (PRR9), and PRR9 promotes the expression of ORE1 while repressing its upstream gene, miR164 [38]. Moreover, RECEPTOR PROTEIN KINASE 1 (RPK1) affects leaf development but depends on leaf age, and inducing RPK1 in old leaves results in enhanced senescence, while the induction of RPK1 in young leaves leads to no signs of senescence but arrested growth [39].

Age-related DNA damage is considered as one of the main triggers of animal senescence [40]. Plants share similarities in DNA-damage-triggered senescence with animals. Endogenous factors such as age can induce leaf senescence, and this may due to increased DNA damage and a decreased DNA repair capacity [41]. The inducible overexpression of I-PpoI restriction endonuclease results in double-strand DNA breaks (DSBs) and leads to an accelerated leaf senescence phenotype [24,25]. DSB-triggered gene expression shares similarities with senescence, and DNA repair genes are also related to leaf senescence, as revealed by transcriptomic data. ATAXIA TELANGIECT ASIA MUTATED (ATM), a human disease related gene [1], is known for its primary DSB signal transducer role [42]. The homolog hit for *Arabidopsis* is involved in leaf senescence and acts as a negative regulator. ATM suppresses the DSB-induced expression of SAGs via the modulation of histone lysine methylation [25].

In this study, we selected 35 disease-associated genes [1] and identified 34 orthologs genes in the *Arabidopsis* and *Populus*. Comparative genomic and molecular docking analysis revealed that orthologs of human-disease-associated genes in plants are involved in regulating leaf senescence.

## 2. Materials and Methods

### 2.1. Identification of Homologous Genes in Arabidopsis and Populus

The *Arabidopsis* genes with similarities to human disease genes were identified from the previous research, an analysis of the genomic sequence of *Arabidopsis* [1]. The name of human disease genes, transcript ID and gene function description in Arabidopsis were displayed in the literature [1] (Table 3 on page 7 (volume page 802), which named ‘Table 3 Arabidopsis genes with similarities to human disease genes’). The homolog genes of *Populus* were identified on the *Populus trichocarpa* v3 database using BLAST, and the most similar transcript and protein sequences were used for further analysis (https://phytozome-next.jgi.doe.gov/ (accessed on 5 September 2022)) [43].

### 2.2. Synteny Analysis, Gene Structure and Phylogenetic Tree Analysis

The protein and annotation files of human (*Homo sapiens*) GRCh38 were downloaded from Ensembl database (https://www.ensembl.org/Homo_sapiens/Info/Index (accessed on 5 January 2023)). The protein and annotation files of *A. thaliana* TAIR10 and *P. trichocarpa* v3 were downloaded from EnsemblPlants database (https://plants.ensembl.org/index.html (accessed on 5 January 2023)). The DIAMOND algorithm [44] was applied for protein sequence alignment between human and *A. thaliana*, as well as human and *P. trichocarpa*, with *E*-value < 1 × 10^−3^ cutoff. Then, the alignment results were visualized in TBtools [45] via Multiple Synteny Plot plugin. The structural maps of the 34 candidate *A. thaliana* and *P. trichocarpa* genes were constructed using the GSDS2.0 tool (http://gsds.gao-lab.org/ (accessed on 5 September 2022)) [46], and the UTR, exon and intron were visualized with different colors. For the phylogenetic tree analysis, the amino acid sequences of the 34 candidate genes were aligned using ClustalX2 tool [47], and then visualized using MEGA-X software with the Maximum-Likelihood algorithm and Bootstrap test of 100 replications [48].

### 2.3. Expression Pattern during Leaf Senescence

The expression values of the 34 genes during the leaf senescence process were extracted from *A. thaliana* [49] and *Populus tomentosa* [50]. The *A. thaliana* data included 14 time points for the leaf development process, tracked from the growth-to-maturation stage (G-to-M; 4–18 d) to the maturation-to-senescence stage (M-to-S; 16–30 d). The *P. tomentosa* data contains 16 time points for autumn leaf senescence process, tracked over the mature stage (M; L1-10), early senescence stage (ES; L11-L13) and later senescence stage (LS; L14-L16). Furthermore, the raw RNA-Seq data of *A. thaliana* and *P. tomentosa* was downloaded from NCBI’s Sequence Read Archive (SRA) database (https://www.ncbi.nlm.nih.gov/sra/ (accessed on 5 September 2022)) [51], with the SRA study accession number PRJNA186843 for *A. thaliana* [49] and PRJNA561520 for *P. tomentosa* [50], and the detailed information was displayed in Supplemental Dataset 1. The ‘SRR’ format data downloaded using SRAToolkit package tool was transformed to ‘fastq’ format via ‘fastq-dump’ command. For data quality control and reads cleaning, the adapter in the reads were removed, and then the low-quality reads (reads with Q_phred_ <= 20 bases account for more than 50% of the entire read) and reads with a ratio of N (N means that the base information cannot be determined) greater than 10% were also removed. Then, the clean reads were aligned to the Arabidopsis genome [52] and *Populus* v3 genome [43] using HISAT2 algorithm, respectively [53]. Transcripts Per Million (TPM) [54] was used to measure gene expression levels and log-transformed values for visualization. The heatmaps were generated by using the ‘pheatmap’ package [55] in R v4.1.2 program. The red and blue color represent the high and low expression levels, respectively.

### 2.4. Construction of Protein–Protein Interaction Network

The online STRING tool (https://cn.string-db.org/ (accessed on 7 December 2022)) was applied for the protein–protein interaction (PPI) data analysis, and then the interaction data were visualized in Cytoscape v3.7. The large circles represent the 34 candidate genes, while the small circles or rectangles represent the potential interacting proteins, and the SAGs are marked in yellow.

### 2.5. Molecular Docking of Drugs and Proteins

The data files of all the proteins used for molecular docking were downloaded from the AlphaFold Protein Structure Database (https://alphafold.com/ (accessed on 7 December 2022)) [56]. The data file of chemical drugs was downloaded from the National Center for Biotechnology Information (NCBI) PubChem Substance Database (https://www.ncbi.nlm.nih.gov/pcsubstance/ (accessed on 7 December 2022)) [57]. The molecular docking was performed using the SwissDock tool (http://www.swissdock.ch/docking (accessed on 7 December 2022)).

### 2.6. Statistical Analyses

GraphPad Prism v8.3.0 software ((Dr. Harvey Motulsky Founder) (Boston, MA 02001, USA) https://www.graphpad.com/scientific-software/prism/ accessed on 7 August 2022) was used to visualize the statistical data, including the exon number, average exon, initial exon, internal exon and terminal exon length.

## 3. Results

### 3.1. Identification of Human Disease-like Genes in A. thaliana and P. trichocarpa

A previous study using BLASTP analysis identified genes with high sequence similarity to human disease genes in the *Arabidopsis* genome [1]. Unlike *A. thaliana*, poplar is a perennial plant. To investigate whether the poplar genome also contains genes with highly similar sequences to human-disease-associated genes, we analyzed the poplar genome using sequence comparisons. To this end, we selected 35 disease-associated genes with high similarity to *Arabidopsis* genes (Table 1). Among these, the *Arabidopsis* homologous gene of both ‘HDL deficiency 1, ABCA1’ and ‘Stargardt’s, ABCA4’ is At2g41700. Thus 34 homologs were identified in the *Arabidopsis* genome. By sequence comparison, we also identified 34 homologs in the poplar genome (*P. trichocarpa* v3.1) (Table 1), with E-values ranging 5.9 × 10^−272^ and 6.9 × 10^−72^, indicating that human-disease-related genes are highly conserved in annual and perennial plants. This table was modified and updated from Table 3 of previous research [1].

### 3.2. Synteny Analysis and Gene Structure Visualization

**Figure 1:**
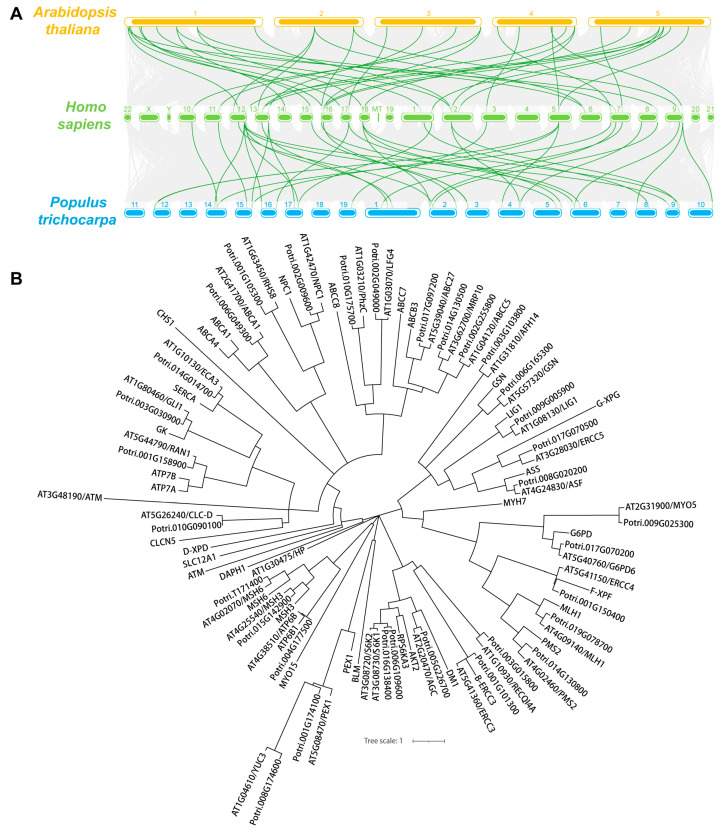
Synteny and phylogenetic tree analysis of 34 human-disease related genes in *A. thaliana* and *P. trichocarpa*. (**A**) The collinearity of 34 gene pairs is emphasized and highlighted in green lines while the synteny blocks between human and *Arabidopsis* or *Populus* are shown in gray lines. (**B**) Phylogenetic tree of 35 human genes and their 34 orthologs in *Arabidopsis* and *Populus*. The amino acid sequence was used for alignment and the human-disease-related genes were marked as green.

**Figure 2:**
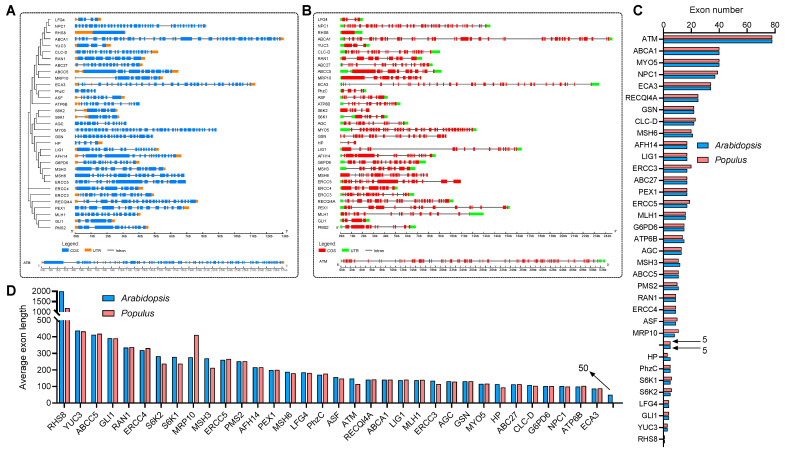
Phylogenetic, gene structure and exon statistical analyses of 34 *Arabidopsis* and *Populus* genes which are similar to human disease genes. (**A**,**B**) The phylogenetic and gene structure of *Arabidopsis* (**A**) and *Populus* (**B**) genes. The genealogical tree was constructed using whole-length amino acid sequences. The sequences were aligned using the ClustalX2 tool and visualized by MEGA-X software. Blue boxes, orange boxes and blacklines indicate exons, UTRs and introns, respectively. (**C**) Exon number statistics of *Arabidopsis* and *Populus* genes, where the arrows indicate the average exon number of each gene on whole-genome level in Arabidopsis and *Populus*, respectively. (**D**) Average exon length statistics of *Arabidopsis* and *Populus* genes, where the arrow indicate the average exon length of each gene on whole-genome level in *Arabidopsis*.

To explore the evolutionary relationship between these genes, a collinear graph was constructed comparing human, *A.thaliana* and *P. trichocarpa* (Figure 1A), and protein pairs highlight in green confirmed the ortholog relationships between human and *A.thaliana*, as well as human and *P. trichocarpa*. The protein alignment results between human and *Arabidopsis* or *Populus* were displayed in Supplemental Dataset 2 and Supplemental Dataset 3, respectively. The phylogenetic tree of the 35 genes in human, and 34 genes in *A.thaliana* and *P. trichocarpa* was constructed based on the amino acid sequence to analyze the ortholog relationship (Figure 1B). The structures of the UTR, exons and introns were also displayed using the Gene Structure Display Server 2.0 (GSDS 2.0) tool (Figure 2A,B). Expect for ROOT HAIR SPECIFIC 8 (RHS8), all other genes contained more than two exons, whether in *Arabidopsis* (Figure 2A) or *Populus* (Figure 2B). The average number of exons was 5.1 and 5.0 in *A.thaliana* [58] and *Homo sapiens* [59], respectively. Surprisingly, 26 (76.5%) of the 34 genes had a greater number of exons than the average number (Figure 2C). Among them, the ATAXIA TELANGIECTASIA-MUTATED GENE (ATM) gene contained the largest number of exons, with 78. More interestingly, we found that the number of exons of the ATM gene in humans is identical to that of the homologs in *A. thaliana* and *P. trichocarpa*. Furthermore, the average exon length of these 34 genes were also analyzed. The exon lengths of the homologous genes in *A. thaliana* and *P. trichocarpa* are similar. Moreover, the average exon length of the 34 candidate genes is much larger than the average on the whole-genome level (50 bp) (Figure 2D). In particular, RHS8 contains a super-sized exon, with 1994 bp.

**Figure 3:**
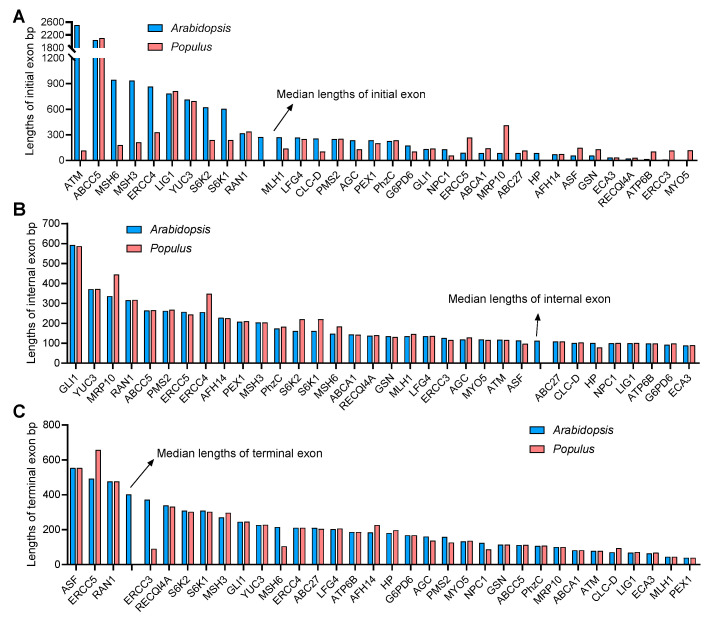
The human-disease-related genes in *Arabidopsis* and *Populus* display longer internal exons but shorter terminal exons. (**A**–**C**) Length of the initial (**A**), internal (**B**) and terminal (**C**) exons of *Arabidopsis* and *Populus* genes. The arrows indicate the medium length of each type of exon.

Furthermore, the lengths of the initial, internal and terminal exons of each gene were also calculated. The initial exon lengths differed greatly between *A. thaliana* and *P. trichocarpa*, while the internal and terminal exon lengths were similar (Figure 3A–C). In particular, the initial exon length of AGC, ATM, CLC-D ERCC4, MLH1, MSH6, MSH3, S6K1 and S6K2 in *A. thaliana* were significantly larger than those in *P. trichocarpa* (Figure 3A).

### 3.3. Gene Expression Analysis Reveals That Most of the Homologous Genes Are Involved in Leaf Senescence Process

**Figure 4:**
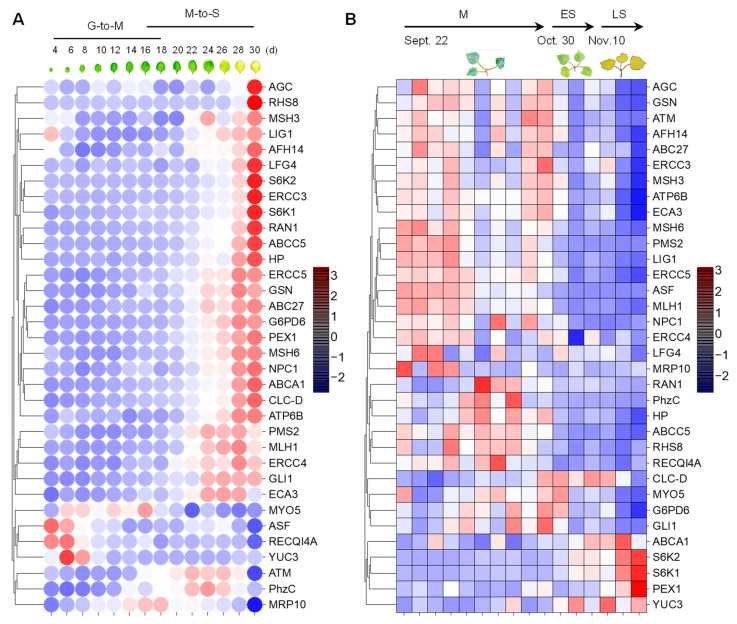
The expression patterns of 34 human-disease-related genes in *Arabidopsis* and *Populus* during the leaf senescence process. (**A**) Heatmap visualization of 34 genes during the leaf senescence process in *Arabidopsis* in a controlled indoor greenhouse environment. (**B**) Expression display of 34 genes during the autumn leaf senescence process of *Populus* in natural outdoor environment. The red/blue color bars indicate the range of expression, and the values are log-transformed from the TPM expression value.

**Figure 5:**
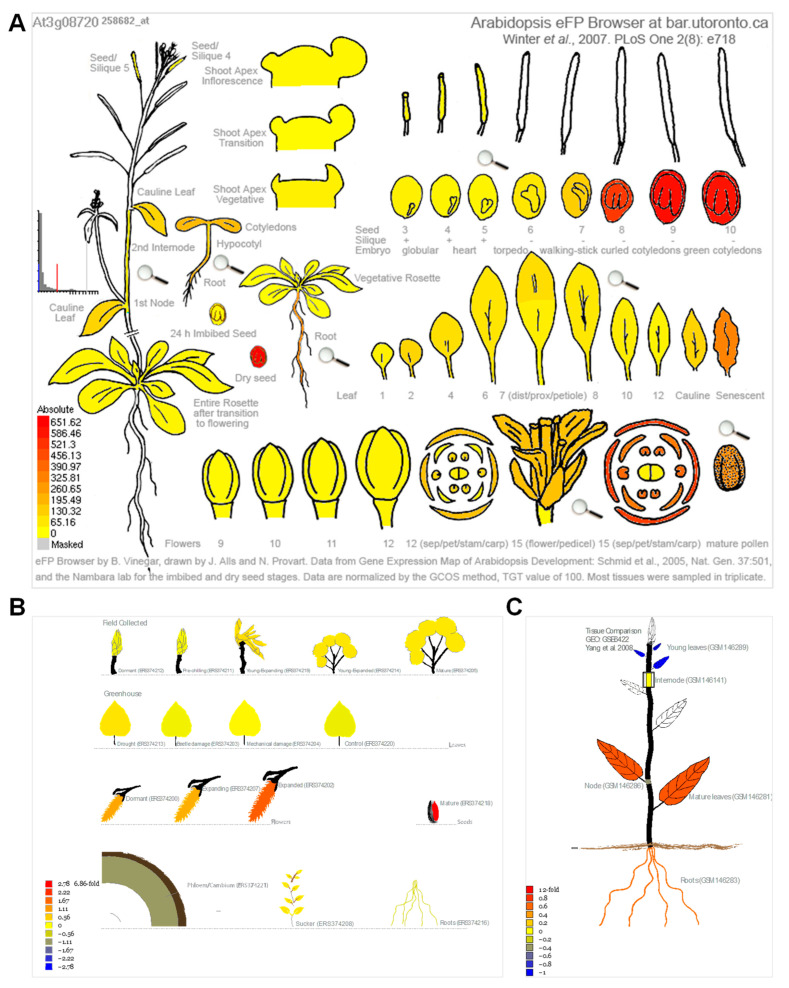
The S6K2 expression in *Arabidopsis* and *Populus*. (**A**) S6K2 expression in *Arabidopsis* according to the *Arabidopsis* electronic fluorescent pictograph (eFP) browser (bar.utoronto.ca/efp/cgi-bin/efpWeb.cgi (accessed on accessed on 13 December 2022)) [60]. (**B**) S6K2 expression in *Populus tremula* according to the popgenie exImage database (https://popgenie.org/eximage?eplant=enable (accessed on accessed on 13 December 2022)) [61]. (**C**) S6K2 expression in different tissues of *P. trichocarpa* according to the popgenie exImage database (https://popgenie.org/eximage?eplant=enable (accessed on accessed on 13 December 2022)) [62].

Because cellular senescence is closely related to human diseases, next, we next investigated whether the homologous genes of disease-associated genes are also involved in the senescence process in plants. To this end, we analyzed the expression pattern of the 34 genes in *Arabidopsis* [49] and *Populus* [50] during leaf senescence. The TPM (transcripts per kilobase of exon model per million mapped reads) value was log-transformed and applied for the data visualization. Gene expression analysis revealed that 27 (79.4%) of the 34 genes were continuously up-regulated during leaf senescence in *Arabidopsis* (Figure 4A). Two genes, ATM and PhzC were up-regulated during early senescence but decreased during later senescence. The expression of four genes, including MYO5, ASF, RECQI4A and YUC3, was continuously decreased during leaf senescence. Interestingly, the peak expression of MRP10 occurred in the G-to-M and M-to-S transitional period of leaf development (Figure 4A). Unexpectedly, the expression levels of most of genes were decreased as leaf aged in poplar. Only 5 (14.7%) of the 34 genes were continuously up-regulated during leaf senescence in *Populus* (Figure 4B). Four of five genes, including ABCA1, S6K1, S6K2 and PEX1, were up-regulated during leaf senescence in *Arabidopsis* and *Populus*. ATM and PhzC showed increased expression patterns that suddenly decreased in the last leaf developmental stage. YUC3 displayed contrasting expression patterns between in *Arabidopsis* and *Populus*. Furthermore, we found that S6K2 is highly expressed in senescent and cauline leaves, according to the *Arabidopsis* electronic fluorescent pictograph (eFP) browser (Figure 5A) [60], a pattern which is similar with the expression in *Populus* (Figure 5B,C) [61,62]. S6K2 shows gradient expression from the apical leaf to the basal leaf stages (Figure 5C) [62]. These results confirm that the expression of S6K2 may be conserved between *Arabidopsis* and *Populus*.

### 3.4. Protein–Protein Interaction Network Analysis Reveals That 34 Homologous Genes Are Integrated with SAGs

**Figure 6:**
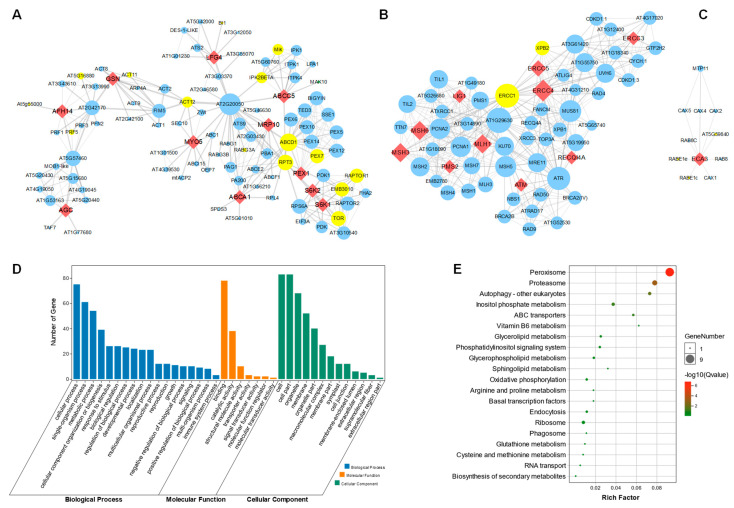
Protein–protein interaction network construction of the candidate genes. (**A**) PPI network centered with LFG4, ABCC5, ABCA1, MRP10, S6K1, S6K2, MYO5, PEX1, GSN, AFH14 and AGC. Red diamond nodes indicate the human disease similarity genes in *Arabidopsis*. Small blue circle nodes are proteins that might interact with these genes. Yellow circle nodes are marked as senescence-associated genes (SAGs). The thickness of the line indicates the possibility of interaction, and a thicker line displays higher possibility of interaction. Larger size of the circle nodes indicate more interactions. (**B**) PPI network centered with LIG1, MSH3, MSH6, PMS2, MLH1, ERCC3, ERCC4, ERCC5, RECQ4A and ATM. (**C**) PPI network that is centered with ECA3. (**D**) GO function enrichment of all genes in (**A**). (**E**) KEGG enrichment of all genes in (**A**).

To analyze the interactions between the 34 homologous genes in *Arabidopsis*, we further constructed the protein–protein interaction (PPI) network. Each of the genes were blasted using the online STRING tool, and the PPI data were further visualized in cytoscape v3.7 software. Interestingly, 11 (32.3%) of the 34 genes formed a first large sub-network (Figure 6A), while 10 (29.4%) of the 34 genes formed second big sub-network (Figure 6B). The ABCA1, LFG4, ABCC5, MRP10, S6K1, S6K2, MYO5, PEX1, GSN, AFH14 and AGC were integrated with 90 other genes in the PPI. Interestingly, 15 of the 90 genes were senescence-associated genes (SAGs), marked in the yellow circle. In addition, LIG1, MSH3, MSH6, PMS2, MLH1, ERCC3, ERCC4, ERCC5, RECQI4A and ATM were integrated with 52 other genes, including 2 SAGs (Figure 6B). ECA3 was integrated integrates with three SAGs, including AT5G59840, RABE1e and RABE1c (Figure 6C). To further explore the detailed pathways of the central genes involved in regulating leaf senescence, we analyzed all these genes in the first sub-network, shown in Figure 6A, via GO (Gene Ontology) and KEGG (Kyoto Encyclopedia of Genes and Genomes) enrichment. We found that the involved genes may participate in regulating the cellular process with binding and catalytic activity functions (Figure 6D). Moreover, these genes are specifically enriched in peroxisome signaling pathways, as well as proteasome, autophagy and inositol-phosphate-metabolism-related processes (Figure 6E), which might provide key clues for further functional study of these human-disease-related genes.

**Figure 7:**
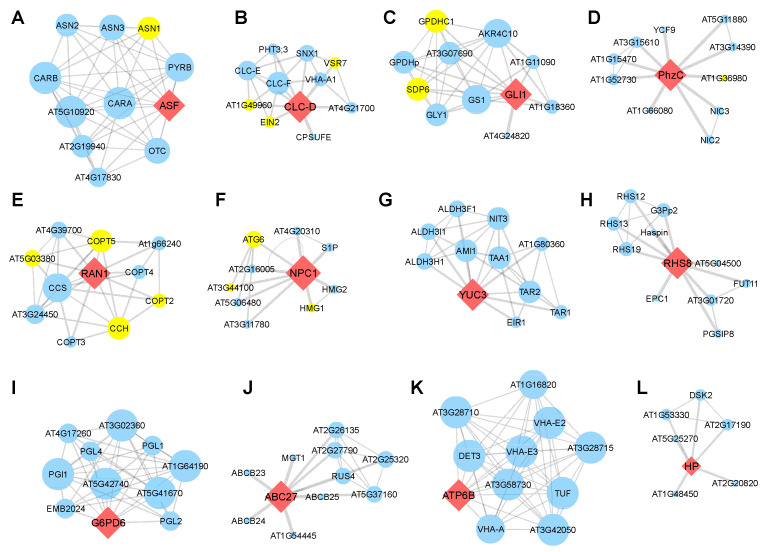
Protein–protein interaction network construction of the candidate genes. (**A**–**L**) PPI network centered with ASF (**A**), CLC-D (**B**), GLI1 (**C**), PhzC (**D**), RAN1 (**E**), NPC1 (**F**), YUC3 (**G**), RHS8 (**H**), G6PD6 (**I**), ABC27 (**J**), ATP6B (**K**) and HP (**L**). Red diamond nodes indicate the human-disease-related genes in *Arabidopsis*. Blue circle nodes are proteins that might interact with these genes. Yellow circle nodes are marked as senescence-associated genes (SAGs). The thickness of the line indicates the possibility of interaction, and a thicker line denotes higher possibility of interaction. Larger circle nodes indicate more interactions.

Meanwhile, 13 genes (38.2%) formed an individual PPI network, itself in performing central role. The ECA3 (Figure 6C), CLC-D (Figure 7B), RAN1 (Figure 7E), and NPC1 (Figure 7F) were found to interact with more than two SAGs. The RAN1 interacts with four SAGs, occupying 40% of the predicted network (Figure 7E). Together, the PPI analyses further suggested that these homologous genes are involved in regulating leaf senescence in *Arabidopsis*.

### 3.5. Molecular Docking Investigation Provides Information and Potential Clues and Approaches for Human Disease Cures by Drug Screening Using the Leaf Senescence Process

Our findings revealed that 27 (79.4%) of the 34 human-disease-related genes were continuously up-regulated during leaf senescence in *Arabidopsis*, which raised the possibility of using the leaf senescence system to study the mechanisms of action of these homologs, which could then be used to unravel the pathogenesis of human diseases and to screen for possible drugs that can be used to treat these diseases. This analysis might increase the value of research on plants such as *Arabidopsis* in understanding human disease states [1,63]. Molecular docking is always used to predict the binding sites of drugs to proteins so as to provide the additional important information regarding the experimental results [64,65]. In the present study, the computational structure modeling of the candidate proteins was downloaded from the AlphaFold Protein Structure Database, and the chemical drug data file was downloaded from the National Center for Biotechnology Information (NCBI) PubChem Substance Database. The molecular docking was performed using SwissDock tool [66,67].

**Figure 8:**
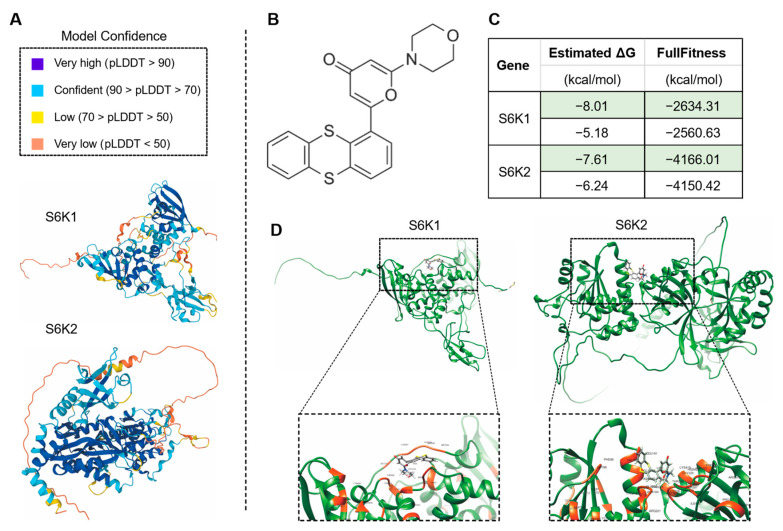
Analysis of the predicted structures and molecular docking of *Arabidopsis* S6K1 and S6K2. (**A**) The predicated structures of S6K1 and S6K2. The data files used for 3D structure visualization were downloaded from the AlphaFold Protein Structure Database. (**B**) Molecular formula of chemical drug KU-55933 (2-Morpholino-6-(thianthren-1-yl)-4H-pyran-4-one). The data file used for molecular docking was downloaded from the PubChem Substance Database. (**C**) Molecular docking of KU-55933 with S6K1 and S6K2. The minimum (marked in green color) and maximum estimated ΔG and FullFitness data are displayed. (**D**) Visualized molecular docking with the minimum estimate ΔG of S6K1 and S6K2, respectively. The orange residues highlight the interaction between the protein and KU-55933.

**Figure 9:**
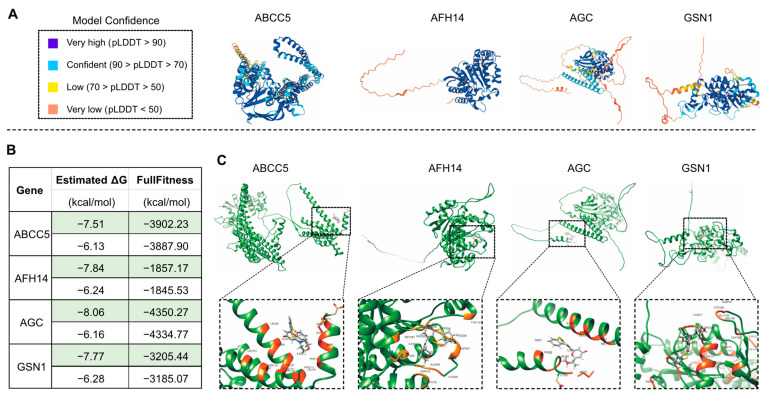
Analysis of the predicted structures and molecular docking of *Arabidopsis* ABCC5, AFH14, AGC and GSN1. (**A**) The predicated structures of ABCC5, AFH14, AGC and GSN1. (**B**) Molecular docking of KU-55933 with ABCC5, AFH14, AGC and GSN1. The minimum (marked in green color) and maximum estimated ΔG and FullFitness data are displayed. (**C**) Visualized molecular docking of the minimum estimated ΔG of ABCC5, AFH14, AGC and GSN1 with KU-55933. The orange residues highlight the interaction between the protein and KU-55933.

The drug KU-55933 was identified as an inhibitor of ATM [68] and was reported to suppresses cell proliferation and induce apoptosis [69], as well as increasing the TMZ responsiveness [70] and sensitizing radioresistant bladder cancer cells [71]. In our study, the molecular docking attempt between *Arabidopsis* ATM and KU-55933 failed. However, we found that several other candidate proteins, including S6K1 and S6K2 (Figure 8), ABCC5, AFH14, AGC and GSN1 (Figure 9), had primary substrate binding pockets on the molecular surface, which might effectively capture KU-55933. The predicted structure of S6K1 shows a high per-residue confidence score (pLDDT) (Figure 8A). KU-55933 (Figure 8B) has great selectivity for ATM but also has selectivity for other related kinases, such as PI3K [69]. There are more than 20 potential binding combinations between KU-55933 and S6K1, with the estimated ΔG varying from −8.01 to −5.18 kcal/mol (Figure 8C). For SK62, KU-55933 has a minimum and maximum estimated ΔG of −7.61 and −6.24, respectively. Therefore, KU-55933 might have selectivity for S6K1 that is greater than that of S6K2. Moreover, the visualization data show the binding site of *Arabidopsis* S6K1, with KU-55933 located in a linearization region, and S6K2, with KU-55933 located at a constricted cleft with loop regions (Figure 8D). In addition, the docking results demonstrated that residue GLY63, as well as LYS237 and THR453 of S6K1, can form hydrogen bonds with KU-55933. Thus, the hydrogen bonds between the amino acids of S6K1 and the oxygen atoms of KU-55933 are the major factors in the complex formation. For S6K2, the two contiguous amino acid regions, ASN184 to LEU191 and PHE383 to ASP389, might form hydrogen bonds with KU-55933. Moreover, KU-55933 might also have selectivity for ABCC5, AFH14, AGC and GSN1 (Figure 9). KU-55933 might bind the initial position of ABCC5 and AGC, with contiguous amino acids regions. Moreover, KU-55933 has greater selectivity for AGC than the other three proteins, depending on the estimated ΔG value (Figure 9B).

**Figure 10:**
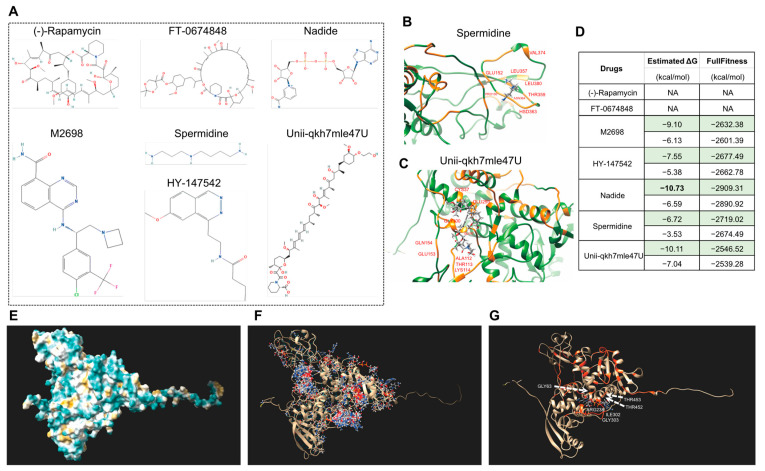
Molecular docking of *Arabidopsis* S6K1 with seven chemicals for drug screening. (**A**) Molecular formula of seven chemical drugs. The data file used for molecular docking was downloaded from the PubChem Substance Database. (**B**,**C**) Visualized molecular docking of S6K1 with spermidine (**B**) and Unii-qkh7mle47U (**C**). The orange residues highlight the interaction between the protein and drug. (**D**) The minimum (marked in green color) and maximum estimated ΔG and FullFitness data are displayed. (**E**) Superposed models of the surface of S6K1. (**F**) All the potential interaction sites of S6K1 with Nadide. (**G**) The docking with minimum estimated ΔG. The orange residues highlight the interaction and the amino acid are marked in white.

Furthermore, we also performed the molecular docking of seven drugs, and some were reported to be related to human disease and senescence [72], including (-)-Rapamycin [73,74], FT-0674848, Nadide [75,76], M2698 [77], Spermidine [78,79], HY-147542, and Unii-qkh7mle47U. The molecular formulae are displayed in Figure 10A. The molecular docking results showed that five of the drugs can bind S6K1 (Figure 10B–D), and Nadide can bind S6K1, with a minimum estimated ΔG value of −10.73, indicating that treatment with nadide will most likely lead to an altered leaf senescence altered phenotype. Moreover, there are more than 20 potential binding sites on the surface of S6K1 (Figure 10E–G), and the visualized docking results exhibited that residue GLY63 as well as ARG238, ILE302, GLY303, THR452 and THR453 of S6K1 can form hydrogen bonds with Nadide. More interestingly, Nadide shares the same analyzed residues as KU-55933, such as GLY63 and THR453, indicating that this region of S6K1 might form a general capture pocket.

### 3.6. Role of Leaf Senescence Research in Understanding Human Disease States

**Figure 11:**
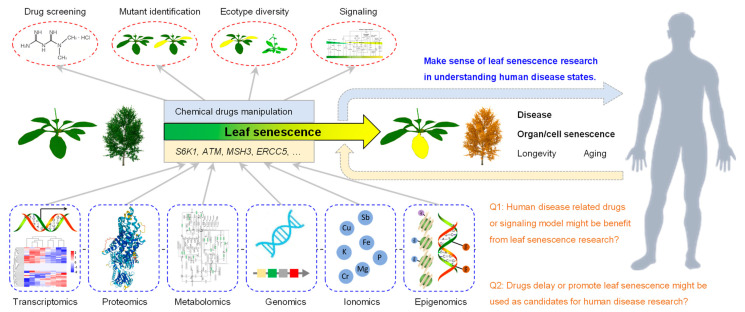
Model for using leaf senescence biological process to study human disease. As 27 of the 34 genes are SAGs, i.e., 79.4%, leaf senescence can be used as a critical biological process to study human disease. Multiple omics methods including transcriptomics, proteomics, metabolomics, genomics, ionomics and epigenomics can be used. Additionally, drug screening, mutant identification, ecotype diversity and signaling can be applied to analyze the gene function. The in-depth analysis of ATM, MSH3, ERCC5, MLH1, PMS2 and other genes might provide important information clue for human disease research.

*A. thaliana* is widely used as a key model organism for plant biology due to its excellent features such as its small genome, rapid reproduction, and multiple ecotypes. The natural process of leaf senescence occurs after growth for 24 days (Figure 5). One can easily observe the leaf senescence phenotype of the 27 up-regulated genes via the genetic mutant. The study of these 27 genes and their roles in regulating leaf senescence, especially the signaling pathways, might provide ideas for this human disease research. Moreover, the development of human-disease-related drugs or signaling pathway models might benefit from transcriptomics, proteomics, metabolomics, genomics, ionomics and epigenomics analyses performed on the genetic material in *Arabidopsis*. Furthermore, drugs delaying or promoting the leaf senescence process through the repression or activation the functions of these 27 genes might also be used as candidates for human disease research (Figure 11).

## 4. Discussion

Plants are traditionally considered to produce food and scientific research using plants generally aimed to render this production process efficient with a high quality. However, it is becoming increasingly clear that plants are invaluable experimental tools that can enable humans to live better [80]. For example, plants such as *Arabidopsis* can be used to understand the molecular mechanisms that underpin human disease states [63]. A high percentage of human-disease-related genes are also present in *Arabidopsis*, as indicated by genome sequence comparison [1,63]. Protein function and cellular processes are conserved between humans and *Arabidopsis*, even though they are seemingly distant species. Some human diseases have been examined using *Arabidopsis*, such as Alzheimer’s and Parkinson’s disease and the neurological disorder Friedreich Ataxia (FRDA). Half of the genes associated with Alzheimer’s disease (AD) [81] have orthologs in *Arabidopsis* based on genetic identification [63]. AtPreP1 (*Arabidopsis* pre-sequence protease 1) and AtPreP2 (*Arabidopsis* pre-sequence protease 2) are orthologs to PITRM1, a human-AD-related protein that degrades Aβ in the human brain mitochondria [82]. AtPrePs share 48% sequence similarity with Human PreP (hPreP). Unstructured small peptides ranging between 10 and 65 amino acids (AA) in length can be digested by AtPreP1 and AtPreP2, and free transit peptides (TP) from chloroplast proteins and pre-signal peptides from mitochondrial proteins are the degradation targets [83,84,85]. Furthermore, the direction and ultimately new findings of human PreP research related to AD are derived from plant PrePs, especially the fundamental research on 3D structural analysis, subcellular localization and substrate specificity [63]. The low abundance (less than 70%) of a mitochondrial iron-binding protein Frataxin (FXN), which is involved in iron–sulfur cluster ([Fe–S]) biosynthesis, leads to a severe neurodegenerative disorder, Friedreich ataxia (FRDA) [86]. A study on *Arabidopsis* frataxin (AtFH) uncovered that a low abundance of FXN causes increased ROS production and high sensitivity to oxidative stress, and this oxidative stress can be counteracted by high levels of nitric oxide (NO) [87]. Consistent with AD, the understanding or treatment of FRDA could also benefit from fundamental research results on *Arabidopsis*.

The previous analysis found that 139 (48%) of 289 human-disease-associated genes were similar to genes in *Arabidopsis*, indicating evolutionary conservation in plants and animals. In this study, we selected 35 disease-associated genes and identified 34 homologous genes in the annual *Arabidopsis* and perennial poplar for the purpose of in-depth analysis. The gene structure and exon analysis revealed that both in *Arabidopsis* and in poplar, these 34 genes had many exons, being well above the average number. These genes have multiple transcripts, which are typical of variable shearing phenomena, mainly exon skipping and intron lagging, and may be a direction for future research. Moreover, 27 of the 34 genes are continuously up-regulated during leaf senescence in *Arabidopsis*, while in *Populus*, this gene number is 5 (Figure 4). One of the possible reasons for this is that *Arabidopsis* was cultured inside a greenhouse, and the growth conditions were controlled. On the other hand, the *Populus* trees grew in a wild field and were subject to various biotic and abiotic stresses. Regarding this specificity, *Arabidopsis* is more suitable for the establishment of leaf-senescence-regulating model that can be used to study human disease. Moreover, we could also conduct environment controlled leaf senescence tracking process in *Populus* for future comparison. The PPI network identified 15 SAGs (Figure 6A), including TARGET OF RAPAMYCIN (TOR) integrated two proteins S6K1 and S6K2 which were up-regulated during leaf senescence in both *Arabidopsis* and *Populus*. TOR has been widely studied in both mammals [88,89,90] and plants [91,92,93,94]. Fundamental research on the human diseases Coffin–Lowry (RPS6KA3) and AKT2, caused by S6K1 and S6K2, respectively, might benefit from TOR, especially in regard to future research directions for TOR focusing on leaf senescence regulation.

Molecular docking is a powerful method of drug-protein screening. The two genes S6K1 and S6K2, which are up-regulated in both Arabidopsis and poplar during leaf senescence, displayed different binding pockets when analyzed with KU-55933 (Figure 8), and this could be further studied through experiments. Moreover, 27 of the 34 genes could be studied by molecular docking for drug screening, and the identified drugs could be applied if they can delay or promote the leaf senescence process. The 27 genes could be genetically studied and manipulated using the excellent features of the small *Arabidopsis* genome, including its rapid reproduction, and multiple ecotypes. The screened small molecule compounds which can alter leaf senescence process might provide good candidates for curing human diseases. Many human diseases arise as a consequence of localized cellular senescence, and if drugs can be developed to directly accelerate the progression of these cells from senescence to apoptosis, this may also provide indications to develop cures for human diseases.

## 5. Conclusions

Our analysis revealed that the similar genes associated with human diseases in *Arabidopsis* and *Populus* display greatly more exon number and longer average exon length than the whole-genome average level. Moreover, our study uncovered that in *Arabidopsis* most of the human disease-related genes were continuously up-regulated during leaf senescence, while in *Populus* most of the human disease-related genes were continuously down-regulated during leaf senescence. ABCA1, S6K1, S6K2 and PEX1, were up-regulated during leaf senescence both in *Arabidopsis* and *Populus*. This study uncovered that human-disease-associated genes in *Arabidopsis* and *Populus* are involved in the process of leaf senescence regulation, which was further confirmed by PPI network and molecular docking analysis. In conclusion, understanding of the leaf senescence fundamental mechanisms might be of central importance to human disease treatment.

## Figures and Tables

**Table 1:** *Arabidopsis* and *Populus* genes with similarities to human disease genes. This table was modified and updated from Table 3 of previous research [1].

Human Disease Gene	*Arabidopsis*	*Populus*	Gene Description	Gene Name
Myotonic dystrophy, DM1	AT2G20470.1	Potri.005G226700.1	AGC (cAMP, cGMP-dependent and protein kinase C)	AGC
Deafness, hereditary, MYO15	AT2G31900.1	Potri.009G025300.1	myosin-like protein XIF, myosin V (MYO5)	MYO5
HDL deficiency 1, ABCA1	AT2G41700.1	Potri.006G049300.1	ATP-binding cassette A1	ABCA1
Stargardt’s, ABCA4	AT2G41700.1	Potri.006G049300.1	ATP-binding cassette A1	ABCA1
Coffin–Lowry, RPS6KA3	AT3G08720.1	Potri.016G138400.1	p70 ribosomal S6 kinase (RPS6KB)	S6K2
AKT2	AT3G08730.1	Potri.006G109600.1	p70 ribosomal S6 kinase (RPS6KB)	S6K1
Xeroderma Pigmentosum, G-XPG	AT3G28030.1	Potri.017G070500.1	DNA excision repair protein ERCC-5 (ERCC5, XPG, RAD2)	ERCC5
Ataxia telangiectasia, ATM	AT3G48190.1	Potri.015G076650.1	ataxia telangiectasia mutated family protein (ATM, TEL1)	ATM
Cystic fibrosis, ABCC7	AT3G62700.1	Potri.014G130500.1	Multidrug resistance-associated protein 10	MRP10
HNPCC, MSH6	AT4G02070.1	Potri.T171400.1	DNA MISMATCH REPAIR PROTEIN MSH6	MSH6
HNPCC, PMS2	AT4G02460.1	Potri.014G130800.1	DNA mismatch repair protein PMS2 (PMS2)	PMS2
HNPCC*, MLH1	AT4G09140.1	Potri.019G078700.2	DNA mismatch repair protein MLH1 (MLH1)	MLH1
Citrullinemia, type I, ASS	AT4G24830.1	Potri.008G020200.1	Argininosuccinate synthase/Citrulline--aspartate ligase	ASF
HNPCC, MSH3	AT4G25540.1	Potri.015G142900.1	DNA mismatch repair protein MSH3 (MSH3)	MSH3
Renal tubul. acidosis, ATP6B1	AT4G38510.1	Potri.004G177500.1	V-type H+-transporting ATPase subunit B (ATPeV1B, ATP6B)	ATP6B
Zellweger, PEX1	AT5G08470.1	Potri.001G174100.4	peroxin-1 (PEX1)	PEX1
Dents, CLCN5	AT5G26240.1	Potri.010G090100.1	H(+)/CL(-) EXCHANGE TRANSPORTER 7	CLC-D
Bare lymphocyte, ABCB3	AT5G39040.1	Potri.017G097200.1	ABC TRANSPORTER B FAMILY MEMBER 27	ABC27
G6PD deficiency, G6PD	AT5G40760.1	Potri.017G070200.1	glucose-6-phosphate 1-dehydrogenase	G6PD6
Xeroderma Pigmentosum, F-XPF	AT5G41150.1	Potri.001G150400.1	DNA excision repair protein ERCC-4 (ERCC4, XPF)	ERCC4
Xeroderma pigment, B-ERCC3	AT5G41360.1	Potri.001G101300.3	DNA excision repair protein ERCC-3 (ERCC3, XPB)	ERCC3
Wilson, ATP7B	AT5G44790.1	Potri.001G158900.1	Cu(+) exporting ATPase/copper-exporting ATPase	RAN1
Finnish amyloidosis, GSN	AT5G57320.1	Potri.006G165300.1	villin/gelsolin (GSN)	GSN
Darier–White, SERCA	AT1G10130.1	Potri.014G014700.2	endoplasmic reticulum-type calcium-transporting ATPase 3	ECA3
Xeroderma Pigmentosum, D-XPD	AT1G03210.1	Potri.010G175700.1	Phenazine biosynthesis PhzC/PhzF protein	PhzC
Hyperinsulinism, ABCC8	AT1G04120.1	Potri.002G255800.4	multidrug resistance-associated protein 5	ABCC5
Immunodeficiency, DNA Ligase 1	AT1G08130.1	Potri.009G005900.2	DNA ligase 1	LIG1
Niemann–Pick, NPC1	AT1G42470.1	Potri.002G009600.2	Niemann-Pick C1 protein (NPC1)	NPC1
Menkes, ATP7A	AT1G63450.1	Potri.001G105300.2	root hair specific 8	RHS8
Fam, cardiac myopathy, MYH7	AT1G04610.1	Potri.008G174600.1	Probable indole-3-pyruvate monooxygenase YUCCA3	YUC3
Glycerol kinase defic, GK	AT1G80460.1	Potri.003G030900.1	Actin-like ATPase superfamily protein	GLI1
Bloom, BLM	AT1G10930.1	Potri.003G015800.1	DNA helicase isolog	RECQl4A
Chediak–Higashi, CHS1	AT1G03070.1	Potri.002G049000.1	Bax inhibitor-1 family protein	LFG4
Bartter’s, SLC12A1	AT1G30475.1	Potri.011G163901.1	hypothetical protein	HP
Diaphanous 1, DAPH1	AT1G31810.1	Potri.003G103800.1	Formin Homology 14	AFH14

## Data Availability

Not applicable.

## Supplementary Materials

The following supporting information can be downloaded at: https://www.mdpi.com/article/10.3390/life13020559/s1. Supplemental Dataset 1. Dataset and sequencing project used in this study; Supplemental Dataset 2. Protein alignment results between human and *Arabidopsis*; Supplemental Dataset 3. Protein alignment results between human and *Populus*.
